# Functional annotation of sixty-five type-2 diabetes risk SNPs and its application in risk prediction

**DOI:** 10.1038/srep43709

**Published:** 2017-03-06

**Authors:** Yiming Wu, Runyu Jing, Yongcheng Dong, Qifan Kuang, Yan Li, Ziyan Huang, Wei Gan, Yue Xue, Yizhou Li, Menglong Li

**Affiliations:** 1 College of Chemistry, Sichuan University, Chengdu 610064, P. R. China; 2 Centre for High Performance Computing, Shenzhen Institutes of Advanced Technology, Chinese Academy of Sciences, Shenzhen, 518055, P. R. China; 3 College of Life Science, Sichuan University, Chengdu 610064, P. R. China; 4 College of Computer Science, Sichuan University, Chengdu 610064, P. R. China

## Abstract

Genome-wide association studies (GWAS) have identified more than sixty single nucleotide polymorphisms (SNPs) associated with increased risk for type 2 diabetes (T2D). However, the identification of causal risk SNPs for T2D pathogenesis was complicated by the factor that each risk SNP is a surrogate for the hundreds of SNPs, most of which reside in non-coding regions. Here we provide a comprehensive annotation of 65 known T2D related SNPs and inspect putative functional SNPs probably causing protein dysfunction, response element disruptions of known transcription factors related to T2D genes and regulatory response element disruption of four histone marks in pancreas and pancreas islet. In new identified risk SNPs, some of them were reported as T2D related SNPs in recent studies. Further, we found that accumulation of modest effects of single sites markedly enhanced the risk prediction based on 1989 T2D samples and 3000 healthy controls. The A_ROC_ value increased from 0.58 to 0.62 by only using genotype score when putative risk SNPs were added. Besides, the net reclassification improvement is 10.03% on the addition of new risk SNPs. Taken together, functional annotation could provide a list of prioritized potential risk SNPs for the further estimation on the T2D susceptibility of individuals.

Type 2 diabetes currently believed to be a complex disease and affects millions of peoples worldwide. While onset and progression of T2D are due to a complex interplay of multiple genetic, epigenetic, environmental and developmental factors^1^. The prevalence of T2D poses a scientific and methodological challenge and appeal to researches in prediction of high-risk subjects.

The incidence of T2D can be prevented substantially through insistently physical and pharmacological interventions in high-risk individuals while receiving forewarning alert from risk predictions. The clinical factors, including BMI index, sex, hypertension, fasting plasma glucose, waist circumstance and family history of diabetes, are frequently used in previous risk models^2^,^3^,^4^,^5^,^6^,^7^,^8^,^9^. These apparent traits are considered to be direct or indirect inducing factors to make individuals susceptible to type 2 diabetes. As a matter of fact, the nature of clinical factors is phenotypes of genomes which are born to maintain individual differences. With the genome wide association studies, more and more common genetic variants are identified having convincing associations with risk of diabetes^10^. These variants may account for onset of obesity and familial forms of diabetes and their discovery promote a dozen of work in predicting individuals at risk of T2D by integrating clinical factors and genetic risk scores in form of summing up the number of risk alleles^11^,^12^. However, the increase in predictive scores is under expectation. It seems that T2D risk prediction at the limit of risk loci detection^13^,^14^. Novel analytic methods and more efficient use of biomarkers are required for more accurate risk models.

Fortunately, due to ongoing advances provided by genome-wide association studies (GWAS) and next generation sequencing analyses, the genomic and epigenetic data enrich the field about understanding functional significance of known risk SNPs^15^. Although the identification of risk SNP is critical in illustrating the relationship between human variants and risk for polygenetic disorders, most risk SNPs reside in large introns or distal to coding exons, which in the past are treated as the junk areas in human genome. However, regulatory elements are confirmed in these gene deserts through massive efforts^16^. Also, it is known that the tag SNPs identified in association study are usually in linkage disequilibrium to surrogate SNPs. They are proxy SNPs for each other, the surrogate SNPs often play the functional role in related to risks, but not the tag SNPs in the GWA study^17^,^18^,^19^. Invoking by recent works which successfully annotated known tag SNPs in several kind of cancers, we conducted functional annotation of 65 known risk SNPs related to type 2 diabetes. To meet the demand of improving the T2D risk model. The identified putative risk SNPs are further employed in risk predictions as new biomarkers (see Fig. 1).

In present work, we extract all linked SNPs in a 1 MB window around the 65 tag SNPs. Further analyses were implemented on the candidate SNPs that are in high LD (r^2^ ≥ 0.5) with tag SNPs and meanwhile overlap exons, transcription stat site (TSS) regions and histone modification related regions. For SNPs in exons, we primarily identified the non-synonymous SNPs which probably shift protein functions. In TSS regions, the linked SNPs were inspected whether they will affect the proximal regulatory elements by generating disruptions on binding of transcription factors (TFs), especially, those related to genes involving in onset of diabetes. For the SNPs locating in histone modification regions, depending on epigenetic data and RNA-seq data of pancreas and pancreas islet, we set up three conditions to sort out SNPs having a high probability of affecting gene regulations and expressions. Among all putative risk SNPs through our functional annotations, some of them were yet reported to have relations with T2D in previous works or have eQTL hits, several of them were analyzed in very recent T2D studies, only a small fraction of them have not been reported to have T2D relations.

All putative risk SNPs in aforementioned analyses are mapped to the GWAS data of 4989 British cohort from WTCCC, the genotyped sites are employed to test the hypothesis that the accumulation of tiny effects of risk SNPs would enhance the risk model. Consequently, the area under the curve (A_ROC_) increases from 0.58 to 0.62 in logistic regression model by only using genotype score. Additionally, the net reclassification improvement (NRI) index is 10.03% by adding the new biomarkers. Our work suggested that the integration of genetic and epigenetic data provides a deeper understanding of known disease-related SNPs, and functional annotations are capable of collecting modest effects of risk SNPs. It may potentially improve the T2D risk models based on clinical factors.

## Methods

### Study population

The WTCCC group devotes massive effort to exploring the utility, design and analyses of genome-wide association studies. Their unflagging effort has provided us available genotype data of over 60,000 individuals, including 2,000 T2D samples^20^. Among them, eleven individuals having duplicated genotypic data are excluded, thus 1989 T2D samples remain in our study. The control group contains 3,000 healthy controls, including 1,500 samples from the 1958 British Birth Cohort and 1,500 samples from the UK Blood Service Control Group. For the genotypes called by BRLMM, it is recommended that those with score >0.5 be treated as no calls. Finally, over 500,000 SNPs related to samples are genotyped.

### Genetic and epigenetic data

The integration genetic and epigenetic data enable a comprehensive functional annotation of risk SNPs, which are considered as the first step in understanding the underlying molecular mechanism of pathogenesis of type 2 diabetes. Here, as many as sixty-five confirmed risk SNPs of T2D are employed (details see Supplementary Table S1). They were employed in a recent T2D risk estimation research^21^.

A key part of our work is to determine whether the LD SNPs affect the gene expression or regulations on genes at their locations. Herein, we concentrate on three regions on the human genome: the exon regions, TSS regions and histone modification areas. The coding exon comes first because they are the determinants of transcription products. The annotations of the exons are obtained from ENCODE V19. This dataset also provide us positions of the transcription start sites of human genome. Based on these TSS positions, 2 kb upstream and downstream of the start site are included as the TSS regions because several works have shown that transcription factors can bind on both side of a start site. The histone modification datasets are provided by Roadmap Epigenomics Project, whose data repository on the NCBI Epigenomics Gateway where the chromatin state datasets are open sources. As we know that epigenetic events contribute to the etiology of diabetes, H3K4me1, H3K4me3 and H3K27ac are selected for gene activation analyses and H3K27me3 is employed for gene repression analysis^22^. Besides, the RNA-seq data of pancreas and pancreas islet are also obtained from NCBI for gene expression analysis. All genomic location information is based on hg19, the data on other genome coordinates is converted to hg19 by LiftOver in UCSC.

### Functional annotation and related tools

The present work initiates from 65 tag SNPs. the putative risk SNPs in linkage disequilibrium with these known risk SNPs are identified by FunciSNP, an R package that allows a population based identification of LD SNPs from 1,000 genome project^23^. FunciSNP had successfully assisted annotation works of risk SNPs associated with several kinds of cancer^17^,^18^,^24^. SNPs involved in coding exons are known to probably cause single amino acid substitutions, which are considered as risk factors related to dysfunction of proteins. Four well-known predictor, poly-phen2, SIFT, PROVEAN and FATHMM, are used to identify the deleterious missense variants^25^,^26^,^27^,^28^. In analyses of SNPs involved in TSS region, we perform in silico search of detecting TF response elements by utilizing FIMO and HOMER, aiming at a full utilization of known TF motifs. In addition, an R/Bioconductor package MotifBreakR measures the extent that how much variant affects the TF response elements^29^. For identification of SNPs may cause dysregulations, we use cufflinks to analyze the RNA-seq data of pancreas and pancreas islet^30^.

### Model construction and measurements

In present work, we quantify the performance of putative risk SNPs from aforementioned functional annotation works in testing the hypothesis that cumulative effects of risk SNPs would elevate predictive scores. The risk score/genotype score is obtained by summing up the number of risk alleles. Logistic regression model is fitted to get the odds ratios of SNPs, including the known risk variants and the putative ones. To assess the performance of new added biomarkers, we calculate the area under curves (A_ROC_) through the overall results, which are generated from a 10-fold cross-validation by logistic regression classification in R. Improvement in the ROC areas represent for the enhancement that new biomarkers bring in. Furthermore, the contributions of putative risk SNPs are more precisely assessed by the net reclassification improvement (NRI) method which tells the numbers of subjects moving to another risk category or remaining in the same risk category while the risk model updates by adding new SNPs. This procedure is operated through R package ‘PredictABEL’.

## Results

### Functional annotation of LD SNPs in exons

132 exon SNPs were identified in linkage disequilibrium (r^2^ > 0.5) with the 65 tag SNPs for type 2 diabetes. Among these linked SNPs, only 37 (28%) locate in the coding region, including 20 synonymous and 17 missense SNPs. While the synonymous SNPs are usually known as benign variants which not change the coding products, the missense SNPs are potential to generate deleterious single amino acids substitutions. We preliminarily estimated the functional effects of these 17 missense SNPs by using four well-established tools SIFT, Polyphen2, PROVEAN and FATHAMM, respectively. The results are summarized in the Table 1. Six SNPs (rs2228603, rs58542926, rs17240268, rs13266634, rs1260326, rs1051334) were regarded as suspected in generating deleterious single amino acid changes which was predicted dysfunction by at least one algorithm. The rs13266634 is firmly associated with decreased insulin release^31^. The effects of rs58542926 could influence the hepatic fibrosis progression in patients with non-alcoholic fatty liver disease (NAFLD)^32^. The rs2228603 is most strongly associated with hepatic triglyceride content (HTGC), an index closely related to fatty liver disease^33^. The rs1260326 is another missense variant related with hyperglycemia, which was also related with liver fat content confirmed in a very recent work^34^. The rs17240268 and rs1051334 were also analyzed in some works but no conclusions were drawn that they are relevant to onset of T2D. For the other exon SNPs, located in the UTR regions, ten have phased genotype information for both T2D samples and healthy controls. We then investigated whether these SNPs involved in the microRNA binding by the integrated resources of miRcode and miRNASNP database^35^,^36^. Three SNPs were identified related to gain/loss target of miRNA. One regulated gene NOTCH2 was reported in pathogenesis of T2D in previous works (see Table 2).

### LD SNPs affect TF binding in Promoter regions

We then investigated those SNPs located in the promoter regions for their effects on the transcript factor binding. 2 kb upstream and downstream of the transcript start site (TSS) was taken as the TSS region which harbors proximal promoters. Through using the FunciSNP by taking TSS regions as the biofeatures, we detected 252 high LD (r^2^ > 0.5) SNPs. These SNPs involving in TF binding sites are likely to alter response elements, further possibly affect regulated genes. Limited by the experimentally verified TF binding motif, two well-established tools, HORMER and FIMO^37^,^38^, were employed to define the binding motif in the promoter regions. The former used build-in known motifs and the later employed TF binding profiles from JASPAR 2016^39^. 188 LD SNPs were found involved in binding sites, among which 125 SNPs related to more than two binding events. Firstly, the activity of SNPs and motifs are simply measured by counting the number of affected motifs and disturbing SNPs in respectively (See details in Supplementary Table S2). On our hypothesis that the active SNPs/motifs are largely considered increasing risks in gene dysregulations. Actually, we have found some cases related to T2D in high ranking SNPs/motifs. For example, Ptf1a^40^, identified as the most heavily affected motif in HOMER, is broadly accepted as a vital TF in pancreases functions. To further assess the effects of LD SNPs on likely transcription factor binding sites, we used motifbreakR to identify the extent that how much information gain or lost in a loci where the alternate allele compares to the reference allele. Through an in-house calculating by R program, we separately obtain effects that the 188 LD SNPs play on HOMER motifs and JASPAR motifs with default setting (See details in Supplementary Tables S3 and S4). Among these LD SNPs in TSS region, only 17 SNPs are genotyped in our GWAS data, thus we analyze these genotyped SNPs. The results from motifbreakR are collectively displayed in Supplementary Table S5. We found all the genotyped SNPs have strong effects on binding more or less, but how much the correlation with the T2D related genes? We next made an analysis on the function of the related genes.

### Functional annotation of TF affected genes

To identify the T2D related genes that may affected by the TF binding, we first collected the nearby genes of the TSS SNPs and analyzed their functions by DAVID^41^. Finally, we got 13 enrichments for representing function of the TSS SNP related genes. The highest enrichment score is 5.29 and it contains only four disease terms directly towards type 2 diabetes (see Supplementary Fig. S1A). The second cluster (enrichment score: 2.02; Supplementary Fig. S1B) tremendously associated with the GO terms about biological process of homeostasis. We took out genes from the top two enrichments (enrichment score >2): CDKAL1, ADIPOQ, WFS1, NOTCH2, MAEA, THADA, PROX1, IGF2BP2, PPARG, ADAM30 and GCKR. Comparing with 65 known risk SNPs related genes, we found that ADIPOQ and ADAM30 are only related to LD SNPs. Actually, both of them are not newly detected T2D related genes. ADIPOQ is expressed in adipose tissue exclusively and it is dysregulated in obesity^42^. ADAM30 is also frequently discussed in T2D researches because it is related to some proxy SNPs in high linkage disequilibrium with risk SNPs. Next, we look up genes in aforementioned two enrichment sets from TF strong related genes in Supplementary Table S5. As a result, only one gene, the PPARG, is hit among T2D high correlated genes. By inspection, it is a vital gene appeared in dozen of T2D researches and it mainly expresses in adipose tissue^43^,^44^. Once it was designed as target of small molecules in curing T2D and obesity. The SNPs strongly affect TF binding and further possibly dysregulate genes are treated as putative high risk SNPs (red one in Supplementary Table S5).

### T2D risk-related SNPs in distal regulatory elements

We have analyzed the T2D LD SNPs in exon and promoter region. Actually, most of LD SNPs do not fall within these areas but in non-coding regions. They don’t have close relation with specify genes or gene correlated promoter regions, which make them not easily to be interpreted. However, still it is possible that a LD SNP laying in distal regulatory element increases T2D risk by activating or repressing gene expressions. To address this issue, we use histone marks to determine which of the LD SNPs are likely to be part of regulatory elements. Four histone modifications for pancreas and pancreas islet are obtained from the NIH Roadmap Epigenome Mapping consortium, including H3K4me1, H3K4me3 and H3K27ac associated with gene activation and H3K27me3 related to gene repression. We broadly retrieved candidate risk SNPs by separately using these eight histone modification datasets as biofeatures in FunciSNP. As a result, 2786 unique SNPs are in high LD (r^2^ > 0.5) with known risk SNPs and 191 of these unique SNPs are successfully genotyped in our T2D GWAS data. Only genotyped SNPs can be utilized in risk model, so we limit our studies to prioritizing the genotyped LD SNPs. Among the SNPs strongly affected TFs, those ultimately regulating expressed genes are considered as high risk SNPs. The prioritizing procedure includes three steps to choose SNPs with high probability in regulating genes. The first step, we inspect the overlapped SNPs involving in distal regulatory element and transcription factor binding sites, 128 of 191 genotyped SNPs affected at least one motif. Secondly, like the procedure in analyzing TSS SNPs, we retrieved the SNPs that have strong effects on binding assessed by motifbreakR. The threshold set to 0.85 to obtain SNPs having strong effects on binding, only seven SNPs are eliminated in this process. The final step, we download the RNA-seq data of pancreas and pancreas-islet from NIH epigenomic roadmap and calculated the FPKM values of genes by using cufflinks (reference genome: RefSeq gene, hg19). 649 genes (FPKM > 1) are considered as expressed genes. Next, we inspect the SNPs whose strongly affected transcript factors targeting at these expressed genes. As a result, 18 SNPs involved in regulating gene expression and two expressed genes (JUN, FOXC1) are affected (see Supplementary Table S6).

### SNPs involved in co-occurrence of histone marks

**Figure 2:**
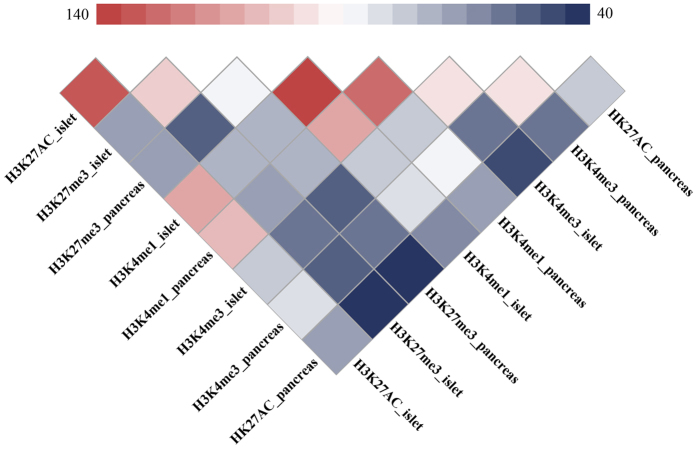
Distributions of genotyped SNPs reside in different colocalization histone marks. It was reported that variants associated with type 2 diabetes are enriched in clustered iselet enhancers. In present work, H3K27me3 marks broadly harbored less overlapped SNPs with the activation marks.

**Figure 3:**
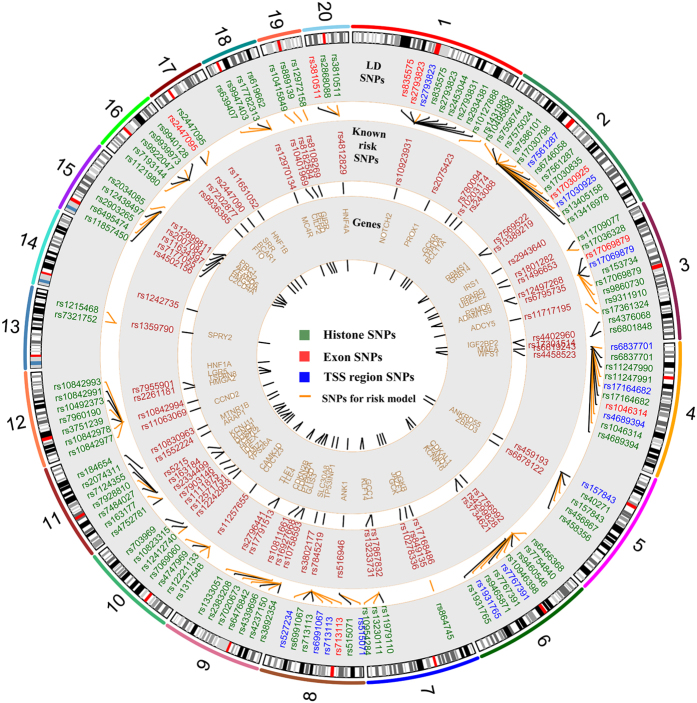
Genome-wide summary of functional annotations of 65 risk SNPs. Detailed map of the locations and annotations associated with risk for type 2 diabetes throughout the human genome. From central circle to outside, each gives the names of proximal genes, tag- or risk- SNPs, correlated SNPs in high LD with risk SNPs. Correlated SNPs pointed by orange short lines are putative risk SNPs through functional annotations.

Histone modifications are known to act in a combinatorial fashion n to determine the overall outcome of gene expression. Besides, it is believed that combinational transcription factor binding existed in human gene transcription. In a recent work, Lorenzo found that five β-cell transcription factors frequently bind to overlapping genomic sites^45^. However, the gene transcription mechanism driven by these combinational effects remain poorly described. How a tiny variant effect is amplified in these combined events is yet to know. Driven by these concerns and support from existing works, we preliminarily discussed SNPs residing in the colocalizations of histone marks. Among the genotyped SNPs related to histone modifications, the numbers of SNPs occupying the overlaps between two histone marks are listed in Fig. 2. In general, the activation related histone modifications harbored more high LD SNPs, which is in consistent with Lorenzo’s finding that risk SNPs associated with type 2 diabetes are enriched in clustered islet enhancers. Besides, the minimized overlapping existed between H3K27me3 and H3K27ac. Once, Reena found that H3K4me3 marks and H3K27me3 marks are usually mutually exclusive in islets^22^. In this work, H3K27me3 marks broadly harbored less overlapped SNPs with the activation marks. Our results supported Reena’s finding, but the underlying mechanism need to be addressed in future works. The similar results can be obtained by using all LD (r^2^ > 0.5) SNPs related to histone modifications, the results are shown in Fig. S2. Considering the localization offsets between tissues, we separately obtained 188 and 166 overlapped SNPs in islet and pancreas. The intersections (163 SNPs) between them are of high confidence affecting multiply histone marks. To further address their potential risks in T2D pathogenesis, we identified those strongly affect motifs in aforementioned works and located in active enhancer clusters which were demonstrated to be regions where are bound by multiple transcription factors in a recent research^45^. Finally, 102 and 16 SNPs remained respectively. The annotation results of histone marks related SNPs are summarized in Supplementary Table S6, rs7901695 and rs2612069 meet all three conditions set in detecting SNPs affecting regulations, rs7901695 had been reported in many T2D researches, rs2612069 was reported as a T2D related SNP in Ballantyne’s very recent work^46^. Besides, three of them (rs1333051, rs1531343, rs1470579) were reported as the confirmed T2D risk SNPs in Europeans in a recent work^10^. We also provide the number of eQTL hits which are obtained from HaploReg for SNPs in histone marks (see details in Supplementary Table S7)^47^. Here we have finished the functional annotation of 65 T2D related risk SNPs, a detailed circos plot (Fig. 3) summarized the locations and annotations of known risk SNPs. In Fig. 3, the potential risk LD SNPs, those pointed by shot orange lines, were further employed by risk model.

### The cumulative effects of risk SNPs

**Figure 4:**
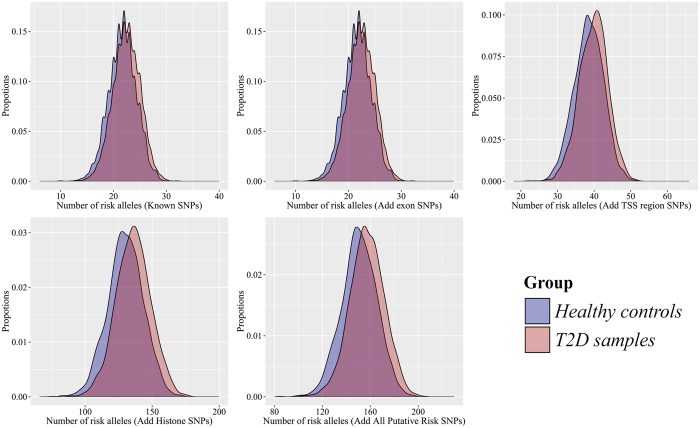
The distribution of genotype score for T2D samples and healthy controls. The degree of separation between two distributions increased along with the increasing number of risk SNPs. In general, T2D samples carry more risk alleles.

So far the underling mechanism of such risk SNPs is unknown; it is believed that single SNPs have too modest effects to lead pathogenic changes. Although the developing GWAS study was increasing the number of identified risk SNPs of T2D, the updating researches find the predicting evaluation is small by using latest risk SNPs. However, the accumulation of such modest effects is considered to widen the implicated process leading to T2D over a lifetime. To address this issue, we count the risk alleles of all the high risk SNPs and compared the deviation of unweighted genotype scores distribution between T2D samples and healthy controls. At different genotypic risk scores by summing up risk alleles of SNPs from different genomic regions, generally, a greater proportion of T2D individuals carry more risk alleles than healthy controls (*t*-test, *P* = 1.5583 × 10^−51^). Besides, the degree of separation between two distributions increased along with the increasing number of risk SNPs (see Fig. 4).

### Association and discrimination using putative risk SNPs

**Figure 5:**
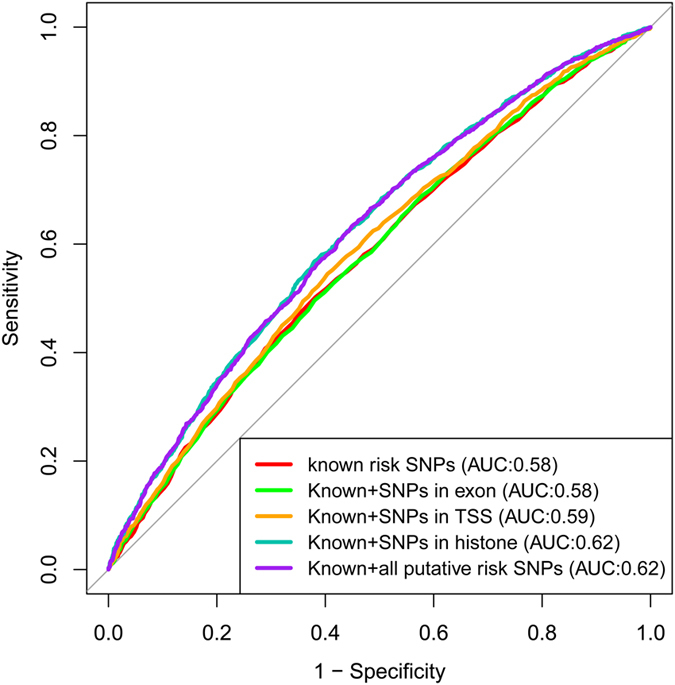
The comparisons of A_ROC_ by adding SNPs from different genomic regions to the known risk SNPs. The additivity of tiny SNP effects markedly improved the prediction.

Unlike previous works adding genotypic risk score to multiple clinical factors to improve risk model, we primarily test the accumulative effects of putative risk SNPs. We used the 17 genotyped SNPs of 65 known risk SNPs as the benchmark. The putative high risk SNPs identified in aforementioned functional annotation works are employed as new biomarkers in logistic regression and discrimination. The model 1 and model 2 were fitted by using benchmark data set and all SNPs respectively. The two models were compared by Chi-squared test (*P* = 2.2 × 10^−16^). The regression coefficients of all putative risk SNPs are estimated from logistic regression model. SNPs with P-value less than 0.05 were listed in Table 3. Next, we obtained the A_ROC_ result from the discrimination based on unweighted genotype score. We compared the performances by integrating benchmark sets with SNPs from different genomic regions. The results are shown in Fig. 5. The A_ROC_ for the known risk SNPs is 0.58. It marginally increased to 0.59 after the addition of exon SNPs and TSS SNPs. The A_ROC_ reached 0.62 when the genotype sore is generated by summing up risk alleles of all SNPs. The addition of histone SNPs almost reached the greatest A_ROC_ value. Although the magnitude of improvement seems small still, it is worth noting that the genotype score is the only feature in the risk model. Moreover, its cumulative effect was detected in prediction, we anticipated that it will enhance the risk model by integrating with multiple well established clinical factors.

### Net reclassification improvement

Furthermore, the contribution of our identified putative risk SNPs are assessed by net reclassification improvement (NRI) measurement^48^, which representing the proportion of individuals that correctly move from one category to another while the new risk biomarkers are added into the regression model, i.e. a T2D individual shift to a higher risk category or a heathy control step into the lower risk categories. The unweighted genotype score including all putative risk variants are added into the reclassification and the results are shown in Table 4. With The risk cutoff of (≥0.2, 0.2–0.4, 0.4–0.6, ≤0.6), we obtain a categorical NRI of 10.03% [95% CI: 6.58%–13.46%; *P* < 0.001], composed of an absent NRI of −0.63% and a present NRI of 10.66%, which indicated that he addition of new risk markers primarily enhanced the identification of T2D samples. For the continuous NRI, which is free from cutoff point, the improvement is 23.48% [95% CI: 17.86%–29.11%; P < 0.001], and for IDI, the improvement is 0.0249 [95% CI: 0.0199–0.0299; *P* < 0.001].

## Discussion

In this study, we conduct a comprehensive functional annotation of 65 tag SNPs known to increase the risk of type 2 diabetes. After a careful inspection on high LD SNPs by integrating genomic data, GWAS data, chip-seq data and RNA-seq data, the putative risk SNPs, with higher probability affecting the pathogenesis of T2D, were sorted out for improving the risk model based on 1989 T2D samples and 3000 healthy controls provided by the Wellcome Trust Case Control Consortium. Through prediction works, the A_ROC,_ NRI, IDI increased in different magnitudes.

Since the GWAS study were confirming more risk SNPs of type 2 diabetes, almost every year, there were new published researches about utilizing expanded risk SNPs to enhance the risk model. However, no matter weighted or unweighted genotype scores, their performances are no good than traditional clinical factors. Although massive efforts had been devoted in utilizing known common variants, it seems that predictive scores reached a plateau in risk allele summing-up fashion. Invoking by recent researches on several kind of cancers, which identified a dozen of disease related SNPs based on known risk SNPs through functional annotation, we plan to exert effects of risk SNPs through a comprehensive search for putative risk SNPs on whole genome and to demonstrate the cumulative risk effect of these SNPs.

Therefore, we analyzed latest 65 T2D related SNPs which were recently used for risk prediction. Integrating with genomic and epigenomic data, we obtained a number of SNPs in high LD with 65 risk SNPs via programme FunciSNP. Next, we separately discussed the LD SNPs in different genomic regions that may lead to T2D in different molecular mechanisms. As a result, we detected relatively new SNPs/genes involving in pathogenesis of T2D comparing to the 65 tag SNPs. Of them, some are identified as T2D related SNPs in previous works, several SNPs are newly discussed after Talmud’s research, but the functions of majorities are needed to be addressed in further works. Purposefully, those were genotyped in our GWAS data (3 in exon, 15 in TSS regions, 112 in histone modified regions), were further employed in regression and discrimination. On the condition of using only one unweighted genotype score on discriminating 1989 T2D samples and 3000 healthy controls. The new biomarkers improved the A_ROC_ from 0.58 to 0.62. After a net reclassification improvement test, the categorical NRI and continuous NRI were 10.03% and 23.48%, respectively.

Meanwhile, there are some limitations to our study. Our GWAS data only contains gender and genotype data of individuals, but without comprehensive clinical factors, we cannot measure the incremental value that genomic data brings to traditional clinical risk model. Although the improvement had been assessed on known risk SNPs based model, we still expect that these cumulative effects would bring us some encouraging results on clinical factor based predictions. The utilization of potential risk SNPs bring another problem, as it is known that the risk allele does not equal to the minor allele, an accurately identification of risk allele could avoid the introduction of bias. However, there is no consensus on how many samples could accurately identify the risk allele. At least, fortunately, the risk alleles, derived from statistical analysis on our T2D and healthy samples, are exactly the same with the known SNPs (*P* = 0.5 × 10^−16^). Moreover, we have noticed that a potential bias could be introduced by summing up risk alleles. E.g., for one T2D sample and one healthy control, the risk allele distribution for SNP A is (1, 0), and for SNP B is (0, 1). When summing up them to obtain the unweighted genotype score (T2D: 1, Healthy: 1), the existed discriminative information will lost. Especially under the condition that without enough known risk SNPs, the majority of individuals harbor intermediate number of risk allele, thus there is a substantial overlap of distributions of risk alleles between T2D samples and healthy controls as reported in previous work^21^ and present work (see Fig. 4). Consequently, the genetic information cannot contribute as much as we expect. Although we have demonstrated that the accumulation of more risk SNPs would give us better results, an effectively utilization of known SNPs could better exert genomic discriminative power.

In conclusion, we have conducted two complementary works: a comprehensive functional annotation of latest 65 known risk SNPs and risk estimation through logistic regression. In combination with genomic, epigenomic and transcriptomic data, we have identified a number of SNPs that are of high probability increase risks to T2D. Although their actual functional mechanisms still need to be addressed, they could be prioritized for analysis in T2D study. Besides, our results in consistent with previous report that risk SNPs enriched in T2D associated enhancers. In the risk prediction, we have demonstrated the hypothesis that the cumulative effects of SNPs could enhance the risk model. The values of A_ROC_ and NRI give consensus results, also, we present our opinion about the utilization of known risk SNPs. We hope this work would invoke motivations in broadening the way to T2D pathogenic analysis and promote the T2D risk predictions which are aided by genomic information.

## Additional Information

**How to cite this article**: Wu, Y. *et al*. Functional annotation of sixty-five type-2 diabetes risk SNPs and its application in risk prediction. *Sci. Rep.* **7**, 43709; doi: 10.1038/srep43709 (2017).

**Publisher's note:** Springer Nature remains neutral with regard to jurisdictional claims in published maps and institutional affiliations.

## Supplementary Material

Supplementary Table S1

Supplementary Table S2

Supplementary Table S3

Supplementary Table S4

Supplementary Table S5

Supplementary Table S6

Supplementary Table S7

Supplementary Materials

## Figures and Tables

**Figure 1:**
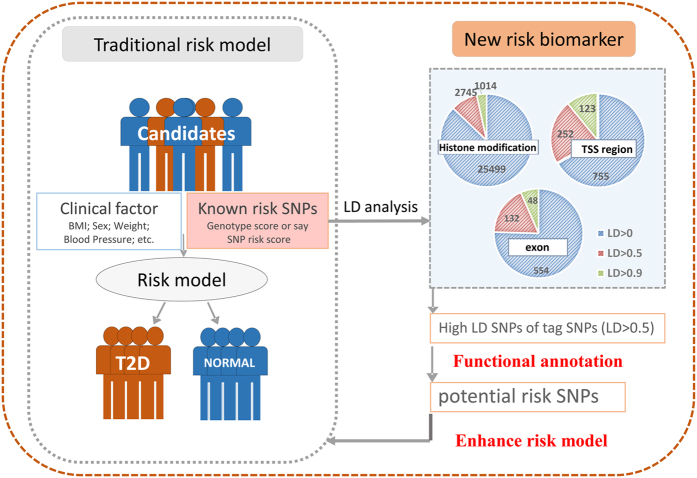
The flowchart of whole procedures, including FunciSNP results on 65 T2D related SNPs. Shown is the flowchart of present work. SNPs in linkage disequilibrium with 65 T2D risk SNPs were obtained by FunciSNP. Functional annotations were executed on high LD SNPs (r^2^ > 0.5) by integrating genomic, epigenetic and transcriptomic data. The putative risks result from annotations were employed for the improvement of risk model.

**Table 1:** Assessment of missense SNPs in exon regions.

SNP	gene	AA change	SIFT	polyphen2	PROVEAN	FATHMM
rs7578597	THADA	T1187A	Tolerated	benign	Tolerated	Tolerated
rs2228603	NCAN	P92S	Tolerated	probably damaging	Tolerated	Tolerated
rs58542926	TM6SF2	E167K	Tolerated	probably damaging	Tolerated	Tolerated
rs2641348	ADA30	L359P	Tolerated	benign	Tolerated	Tolerated
rs11073964	VP33B	G514S	Tolerated	benign	Tolerated	Tolerated
rs56200889	ARAP1	Q1047E	Tolerated	benign	Tolerated	Tolerated
rs17240268	AMPN	A311V	Tolerated	possibly damaging	Tolerated	Tolerated
rs13266634	ZNT8	R276W	Tolerated	NA	NA	Damaging
rs1801212	WFS1	V333I	Tolerated	benign	Tolerated	Tolerated
rs1801214	WFS1	N500K	Tolerated	NA	Tolerated	Tolerated
rs734312	WFS1	R611H	Tolerated	benign	Tolerated	Tolerated
rs5219	IRK11	K23E	Tolerated	benign	NA	Tolerated
rs757081	NUCB2	Q338E	Tolerated	benign	Tolerated	Tolerated
rs757110	ABCC8	A1369S	Tolerated	benign	Tolerated	Tolerated
rs2276904	UVSSA	R391H	Tolerated	benign	Tolerated	Tolerated
rs1260326	GCKR	L446P	Tolerated	possibly damaging	Tolerated	Tolerated
rs1051334	TSPAN8	S213A	Damaging	benign	Tolerated	Tolerated

SNPs potentially disrupting the functions of proteins were assessed by four well-known predictors.

**Table 2:** MicroRNA target SNPs.

SNP	gene	miRNA_id	location
rs3810511	GDAP1L1	miR-423/486/3184/4688	3′UTR
rs17069879	PRICKLE2	miR-582/320e	3′UTR
rs835575	NOTCH2	miR-5590	3′UTR

13698345156. The genotyped SNPs located in exons and involved in microRNA targets.

**Table 3:** Odds ratio and 95% CI of putative risk SNPs (P < 0.05).

SNPs	Categories	Odds Ratio (95% CI)	P value	Nearest gene
rs780094	Known risk SNP	1.66 (1.02–2.75)	4.45E-02	GCKR
rs10203174	Known risk SNP	1.21 (1.04–1.39)	1.10E-02	THADA
rs1496653	Known risk SNP	1.33 (1.01–1.76)	4.46E-02	UBE2E2
rs13233731	Known risk SNP	1.55 (1.03–2.36)	3.63E-02	KLF14
rs5215	Known risk SNP	1.12 (1.01–1.25)	2.79E-02	KCNJ11
rs2793823	TSS region SNP	0.77 (0.61–0.98)	3.26E-02	ADAM30
rs713113	TSS region SNP	1.17 (1.06–1.29)	1.49E-03	CCNE2
rs9311910	Histone modification	0.77 (0.59–0.98)	3.78E-02	MIR548A2
rs11857450	Histone modification	1.41 (1.10–1.81)	7.42E-03	HMG20A
rs1993669	Histone modification	1.89 (1.11–3.39)	2.40E-02	HMGA2
rs7575024	Histone modification	1.16 (1.02–1.32)	2.42E-02	THADA
rs713113	Histone modification	1.19 (1.08–1.30)	2.00E-04	CCNE2
rs11178602	Histone modification	2.66 (1.15–7.80)	4.12E-02	TSPAN8
rs9939973	Histone modification	1.18 (1.05–1.34)	6.96E-03	FTO
rs2260671	Histone modification	1.49 (1.00–2.23)	4.96E-02	HMGA2
rs2868093	Histone modification	1.51 (1.07–2.16)	2.10E-02	R3HDML
rs5018648	Histone modification	0.46 (0.24–0.84)	1.42E-02	WFS1

The ORs (95% CIs) and P values for type 2 diabetes were calculated using logistic regression analysis in 1989 T2D samples and 3000 healthy controls.

**Table 4:** The net reclassification improvement results.

Predicted risk	Reclassified predicted risk (plus new SNPs)				Reclassified number		Net correctly reclassified
	<20%	20 to <40%	40 to <60%	≥60%	Increase risk	Decrease risk	
Healthy controls							
<20%	2	0	0	0	539	520	−0.63%
20 to <40%	80	1276	486	10			
40 to <60%	3	432	663	43			
	0	0	5	0			
T2D controls							
<20%	1	0	0	0	497	285	10.65%
20 to <40%	24	557	390	18			
40 to <60%	1	251	647	89			
≥60%	0	1	8	2			
Net reclassification improvement [95% CI]						10.03% [6.58%–13.47% ]	

Number of subjects reclassified to higher or lower risk categories after the addition of new biomarkers. The improvement classification rates are 10.65% and −0.63% for T2D subjects and healthy controls, respectively, with a total improvement rate of 10.02% (10.65%–0.63%).

## References

[b1] WC K. . Reduction in the Incidence of T2DM with Lifestyle Intervention or Metformin. New England Journal of Medicine 346, 393–403 (2002). 11832527 10.1056/NEJMoa012512 PMC1370926

[b2] Meigs J. B. . Genotype score in addition to common risk factors for prediction of type 2 diabetes. New England Journal of Medicine 359, 2208–2219 (2008). 19020323 10.1056/NEJMoa0804742 PMC2746946

[b3] Van H. M. . Predicting type 2 diabetes based on polymorphisms from genome-wide association studies: a population-based study. Diabetes 57, 3122–3128 (2008). 18694974 10.2337/db08-0425 PMC2570410

[b4] Weedon M. N. . Combining Information from Common Type 2 Diabetes Risk Polymorphisms Improves Disease Prediction. Plos Medicine 3, 1877–1882 (2006). 10.1371/journal.pmed.0030374 PMC1584415 17020404

[b5] Herder C., Kowall B., Tabak A. G. & Rathmann W. The potential of novel biomarkers to improve risk prediction of type 2 diabetes. Diabetologia 57, 16–29 (2014). 24078135 10.1007/s00125-013-3061-3

[b6] Fontaine-Bisson B. . Evaluating the discriminative power of multi-trait genetic risk scores for type 2 diabetes in a northern Swedish population. Diabetologia 53, 2155–2162 (2010). 20571754 10.1007/s00125-010-1792-y PMC2931645

[b7] de Miguel-Yanes J. M. . Genetic risk reclassification for type 2 diabetes by age below or above 50 years using 40 type 2 diabetes risk single nucleotide polymorphisms. Diabetes Care 34, 121–125 (2011). 20889853 10.2337/dc10-1265 PMC3005447

[b8] M’t Hart L. . Combined risk allele score of eight type 2 diabetes genes is associated with reduced first-phase glucose-stimulated insulin secretion during hyperglycemic clamps. Diabetes 59, 287–292 (2010). 19808892 10.2337/db09-0736 PMC2797935

[b9] Weedon M. N. . Combining information from common type 2 diabetes risk polymorphisms improves disease prediction. PLoS Med 3, e374 (2006). 17020404 10.1371/journal.pmed.0030374 PMC1584415

[b10] Fuchsberger C. . The genetic architecture of type 2 diabetes. Nature (2016). 10.1038/nature18642 PMC5034897 27398621

[b11] Müller B. . Improved prediction of complex diseases by common genetic markers: state of the art and further perspectives. Human Genetics 135, 259–272 (2016). 26839113 10.1007/s00439-016-1636-z PMC4759222

[b12] Wang X. . Genetic markers of type 2 diabetes: Progress in genome-wide association studies and clinical application for risk prediction. Journal of diabetes 8, 24–35 (2016). 26119161 10.1111/1753-0407.12323

[b13] Keating B. J. Advances in risk prediction of type 2 diabetes: integrating genetic scores with framingham risk models. Diabetes 64, 1495–1497 (2015). 25908872 10.2337/db15-0033 PMC4407858

[b14] Vassy J. L. . Polygenic Type 2 Diabetes Prediction at the Limit of Common Variant Detection. Diabetes 63, 2172–2182 (2014). 24520119 10.2337/db13-1663 PMC4030114

[b15] Morris A. P. . Large-scale association analysis provides insights into the genetic architecture and pathophysiology of type 2 diabetes. Nature genetics 44, 981 (2012). 22885922 10.1038/ng.2383 PMC3442244

[b16] Zentner G. E. & Scacheri P. C. The chromatin fingerprint of gene enhancer elements. Journal of Biological Chemistry 287, 30888–30896 (2012). 22952241 10.1074/jbc.R111.296491 PMC3438921

[b17] Yao L., Tak Y. G., Berman B. P. & Farnham P. J. Functional annotation of colon cancer risk SNPs. Nature Communications 5, 5114–5114 (2014). 10.1038/ncomms6114 PMC4200523 25268989

[b18] Hazelett D. J. . Comprehensive Functional Annotation of 77 Prostate Cancer Risk Loci. Plos Genetics 10, 229–231 (2014). 10.1371/journal.pgen.1004102 PMC3907334 24497837

[b19] Hall T. O. . Risk prediction for complex diseases: application to Parkinson disease. Genetics in Medicine Official Journal of the American College of Medical Genetics 15, 361–367 (2013). 23222663 10.1038/gim.2012.109 PMC3687522

[b20] Burton P. R. . Genome-wide association study of 14,000 cases of seven common diseases and 3,000 shared controls. Nature 447, 661–678 (2007). 17554300 10.1038/nature05911 PMC2719288

[b21] Talmud P. J. . Sixty-Five Common Genetic Variants and Prediction of Type 2 Diabetes. Diabetes 64, 1830–1840 (2014). 25475436 10.2337/db14-1504 PMC4407866

[b22] Bhandare R. . Genome-wide analysis of histone modifications in human pancreatic islets. Genome Research 20, 428–433 (2010). 20181961 10.1101/gr.102038.109 PMC2847745

[b23] Coetzee S. G., Rhie S. K., Berman B. P., Coetzee G. A. & Noushmehr H. FunciSNP: an R/bioconductor tool integrating functional non-coding data sets with genetic association studies to identify candidate regulatory SNPs. Nucleic Acids Research 40, e139 (2012). 22684628 10.1093/nar/gks542 PMC3467035

[b24] Rhie S. K. . Comprehensive Functional Annotation of Seventy-One Breast Cancer Risk Loci. Plos One 8, e63925–e63925 (2012). 10.1371/journal.pone.0063925 PMC3661550 23717510

[b25] Adzhubei I. A. . A method and server for predicting damaging missense mutations. Nature Methods 7, 248–249 (2010). 20354512 10.1038/nmeth0410-248 PMC2855889

[b26] Kumar P., Henikoff S. & Ng P. C. Predicting the effects of coding non-synonymous variants on protein function using the SIFT algorithm. Nature Protocol 4, 1073–1082 (2009). 10.1038/nprot.2009.86 19561590

[b27] Choi Y. & Chan A. P. PROVEAN web server: a tool to predict the functional effect of amino acid substitutions and indels. Bioinformatics 31, 214–215 (2015). 10.1093/bioinformatics/btv195 PMC4528627 25851949

[b28] Shihab H. A. . Predicting the functional, molecular, and phenotypic consequences of amino acid substitutions using hidden Markov models. Human mutation 34, 57–65 (2013). 23033316 10.1002/humu.22225 PMC3558800

[b29] Coetzee S. G., Coetzee G. A. & Hazelett D. J. MotifbreakR: an R/Bioconductor package for predicting variant effects at transcription factor binding sites. Bioinformatics 31, 3847–3849 (2015). 26272984 10.1093/bioinformatics/btv470 PMC4653394

[b30] C T. . Transcript assembly and quantification by RNA-Seq reveals unannotated transcripts and isoform switching during cell differentiation. Nature Biotechnology 28, 511–515 (2010). 10.1038/nbt.1621 PMC3146043 20436464

[b31] Cauchi S. . Meta-analysis and functional effects of the SLC30A8 rs13266634 polymorphism on isolated human pancreatic islets. Molecular Genetics & Metabolism 100, 77–82 (2010). 20138556 10.1016/j.ymgme.2010.01.001

[b32] Liu Y. L. . TM6SF2 rs58542926 influences hepatic fibrosis progression in patients with non-alcoholic fatty liver disease. Nature Communications 5, 4309–4309 (2014). 10.1038/ncomms5309 PMC4279183 24978903

[b33] Gorden A. . Genetic Variation at NCAN Locus Is Associated with Inflammation and Fibrosis in Non. Human Heredity 75, 34–43 (2013). 23594525 10.1159/000346195 PMC3864002

[b34] Petit J. M. . GCKR polymorphism influences liver fat content in patients with type 2 diabetes. Acta Diabetologica 53, 1–6 (2016). 25976242 10.1007/s00592-015-0766-4

[b35] Jeggari A., Marks D. S. & Larsson E. miRcode: a map of putative microRNA target sites in the long non-coding transcriptome. Bioinformatics 28, 2062–2063 (2012). 22718787 10.1093/bioinformatics/bts344 PMC3400968

[b36] Gong J. . An update of miRNASNP database for better SNP selection by GWAS data, miRNA expression and online tools. Database the Journal of Biological Databases & Curation 2015 (2015). 10.1093/database/bav029 PMC4397995 25877638

[b37] Grant C. E., Bailey T. L. & Noble W. S. FIMO: scanning for occurrences of a given motif. Bioinformatics 27, 1017–1018 (2011). 21330290 10.1093/bioinformatics/btr064 PMC3065696

[b38] Heinz S. . Simple Combinations of Lineage-Determining Transcription Factors Prime cis -Regulatory Elements Required for Macrophage and B Cell Identities. Molecular Cell 38, 576–589 (2010). 20513432 10.1016/j.molcel.2010.05.004 PMC2898526

[b39] Mathelier A. . JASPAR 2014: an extensively expanded and updated open-access database of transcription factor binding profiles. Nucleic Acids Research 42, 142–147 (2014). 10.1093/nar/gkt997 PMC3965086 24194598

[b40] Houghton J. A. . Isolated pancreatic aplasia due to a hypomorphic PTF1A mutation. Diabetes (2016). 10.2337/db15-1666 PMC5001172 27284104

[b41] Huang D. W., Sherman B. T. & Lempicki R. A. Systematic and integrative analysis of large gene lists using DAVID bioinformatics resources. Nature Protocol 4, 44–57 (2009). 10.1038/nprot.2008.211 19131956

[b42] Hu E., Liang P. & Spiegelman B. M. AdipoQ is a novel adipose-specific gene dysregulated in obesity. Journal of Biological Chemistry 271, 10697–10703 (1996). 8631877 10.1074/jbc.271.18.10697

[b43] Chauhan G. . Impact of Common Variants of PPARG, KCNJ11, TCF7L2, SLC30A8, HHEX, CDKN2A, IGF2BP2, and CDKAL1 on the Risk of Type 2 Diabetes in 5,164 Indians. Diabetes 59, 2068–2074 (2010). 20424228 10.2337/db09-1386 PMC2911051

[b44] Black M. H. . Variation in PPARG is associated with longitudinal change in insulin resistance in Mexican Americans at risk for type 2 diabetes. Journal of Clinical Endocrinology & Metabolism 100, 1187–1195 (2015). 25584717 10.1210/jc.2014-3246 PMC4333029

[b45] Pasquali L. . Pancreatic islet enhancer clusters enriched in type 2 diabetes risk–associated variants. Nature Genetics 46, 136–143 (2014). 24413736 10.1038/ng.2870 PMC3935450

[b46] Ballantyne R. L. . Genome-wide interrogation reveals hundreds of long intergenic noncoding RNAs that associate with cardiometabolic traits. Human Molecular Genetics (2016). 10.1093/hmg/ddw154 PMC5181595 27288454

[b47] Ward L. D. & Kellis M. HaploReg v4: systematic mining of putative causal variants, cell types, regulators and target genes for human complex traits and disease. Nucleic Acids Research 44, D877–D881 (2015). 26657631 10.1093/nar/gkv1340 PMC4702929

[b48] Tam C. H. T. . Use of Net Reclassification Improvement (NRI) Method Confirms The Utility of Combined Genetic Risk Score to Predict Type 2 Diabetes. Plos One 8, 1524–1528 (2013). 10.1371/journal.pone.0083093 PMC3869744 24376643

