# Supplementary material for: Functional annotation of sixty-five type-2 diabetes risk SNPs and its application in risk prediction
Source: Sci Rep. 2017 Mar 6;7:43709. doi: 10.1038/srep43709 (PMC5337961; doi:10.1038/srep43709)
Supplement: Supplementary Table S1 [file srep43709-s1.doc]

**Table S1 details of 65 known risk SNPs used in analysis**

| **SNP Rs id** | **CHR** | **Chr position (hg19)** | **Gene** | **Risk allele/other** |
| --- | --- | --- | --- | --- |
| rs10923931 | 1 | 17,408,630 | NOTCH2 | T/G |
| rs2075423 | 1 | 2,847,069 | PROX1 | G/T |
| rs780094 | 2 | 60,568,745 | GCKR | C/T |
| rs10203174 | 2 | 55,806,751 | THADA | C/T |
| rs243088 | 2 | 41,519,248 | BCL11A | T/A |
| rs7569522 | 2 | 27,741,237 | RBMS1 | A/G |
| rs13389219 | 2 | 28,196,413 | GRB14 | C/T |
| rs2943640 | 2 | 94,462,882 | IRS1 | C/A |
| rs1801282 | 3 | 23,196,040 | PPARG | C/G |
| rs1496653 | 3 | 80,717,156 | UBE2E2 | A/G |
| rs12497268 | 3 | 23,454,790 | PSMD6 | G/C |
| rs6795735 | 3 | 72,433,098 | ADAMTS9 | C/T |
| rs11717195 | 3 | 12,393,125 | ADCY5 | T/C |
| rs4402960 | 3 | 90,345,335 | IGF2BP2 | T/G |
| rs17301514 | 3 | 214,154,719 | ST64GAL1 | A/G |
| rs6819243 | 4 | 66,212,318 | MAEA | T/C |
| rs4458523 | 4 | 1,696,849 | WFS1 | G/T |
| rs459193 | 5 | 2,298,974 | ANKRD55 | G/A |
| rs6878122 | 5 | 84,308,948 | ZBED3 | G/A |
| rs7756992 | 6 | 227,093,585 | CDKAL1 | G/A |
| rs4299828 | 6 | 39,304,211 | ZFAND3 | A/G |
| rs3734621 | 6 | 118,185,025 | KCNK16 | C/A |
| rs17168486 | 7 | 38,177,667 | DGKB | T/C |
| rs849135 | 7 | 185,511,687 | JAZF1 | G/A |
| rs10278336 | 7 | 6,289,986 | GCK | A/G |
| rs17867832 | 7 | 62,383,155 | GCC1 | T/G |
| rs13233731 | 7 | 42,989,267 | KLF14 | G/A |
| rs516946 | 8 | 64,705,365 | ANK1 | C/T |
| rs7845219 | 8 | 1,293,245 | TP53INP1 | T/C |
| rs3802177 | 8 | 76,427,311 | SLC30A8 | G/A |
| rs10758593 | 9 | 77,832,762 | GLIS3 | A/G |
| rs16927668 | 9 | 75,247,245 | PTPRD | T/C |
| rs10811661 | 9 | 161,346,447 | CDKN2A/B | T/C |
| rs17791513 | 9 | 20,679,709 | TLE4 | A/G |
| rs2796441 | 9 | 95,937,502 | TLE1 | G/A |
| rs11257655 | 10 | 114,758,349 | CDC123/CAMK1D | T/C |
| rs12242953 | 10 | 71,433,293 | VPS26A | G/A |
| rs12571751 | 10 | 46,158,513 | ZMIZ1 | A/G |
| rs1111875 | 10 | 33,909,710 | HHEX/IDE | C/T |
| rs7903146 | 10 | 53,819,169 | TCF7L2 | T/C |
| rs2334499 | 11 | 43,690,030 | DUSP8 | T/C |
| rs163184 | 11 | 44,245,363 | KCNQ1 | G/T |
| rs5215 | 11 | 19,407,718 | KCNJ11 | C/T |
| rs1552224 | 11 | 4,292,083 | ARAP1(CENTD2) | A/C |
| rs10830963 | 11 | 22,134,094 | MTNR1B | G/C |
| rs11063069 | 12 | 92,708,710 | CCND2 | G/A |
| rs10842994 | 12 | 27,965,150 | KLHDC5 | C/T |
| rs2261181 | 12 | 120,517,959 | HMGA2 | T/C |
| rs7955901 | 12 | 4,374,373 | TSPAN8/LGR5 | C/T |
| rs12427353 | 12 | 12,307,894 | HNF1A(TCF1) | G/C |
| rs1359790 | 13 | 80,432,222 | SPRY2 | G/A |
| rs4502156 | 15 | 36,102,381 | C2CD4A | T/C |
| rs7177055 | 15 | 123,082,398 | HMG20A | A/G |
| rs11634397 | 15 | 70,865,342 | ZFAND6 | G/A |
| rs2007084 | 15 | 64,090,363 | AP3S2 | G/A |
| rs12899811 | 15 | 80,942,631 | PRC1 | G/A |
| rs9936385 | 16 | 91,544,076 | FTO | C/T |
| rs7202877 | 16 | 57,884,750 | BCAR1 | T/G |
| rs2447090 | 17 | 130,437,689 | SRR | A/G |
| rs11651052 | 17 | 165,528,876 | HNF1B(TCF2) | A/G |
| rs12970134 | 18 | 8,369,533 | MC4R | A/G |
| rs10401969 | 19 | 14,898,282 | CILP2 | C/T |
| rs8182584 | 19 | 186,613,409 | PEPD | T/G |
| rs8108269 | 19 | 81,905,590 | GIPR | G/T |
| rs4812829 | 20 | 126,996,837 | HNF4A | A/G |
