# Supplementary material for: Functional annotation of sixty-five type-2 diabetes risk SNPs and its application in risk prediction
Source: Sci Rep. 2017 Mar 6;7:43709. doi: 10.1038/srep43709 (PMC5337961; doi:10.1038/srep43709)
Supplement: Supplementary Materials [file srep43709-s8.pdf]

Supplementary Materials For

Functional annotation of sixty-five type-2 diabetes risk SNPs and its application in risk prediction

Yiming Wu, Runyu Jing, Yongcheng Dong, Qifan Kuang, Yan Li, Ziyan Huang, Wei Gan, Yue Xue, Yizhou Li, Menglong Li

Figure S1 the top 2 enrichments of genes related to LD SNPs in TSS regions.

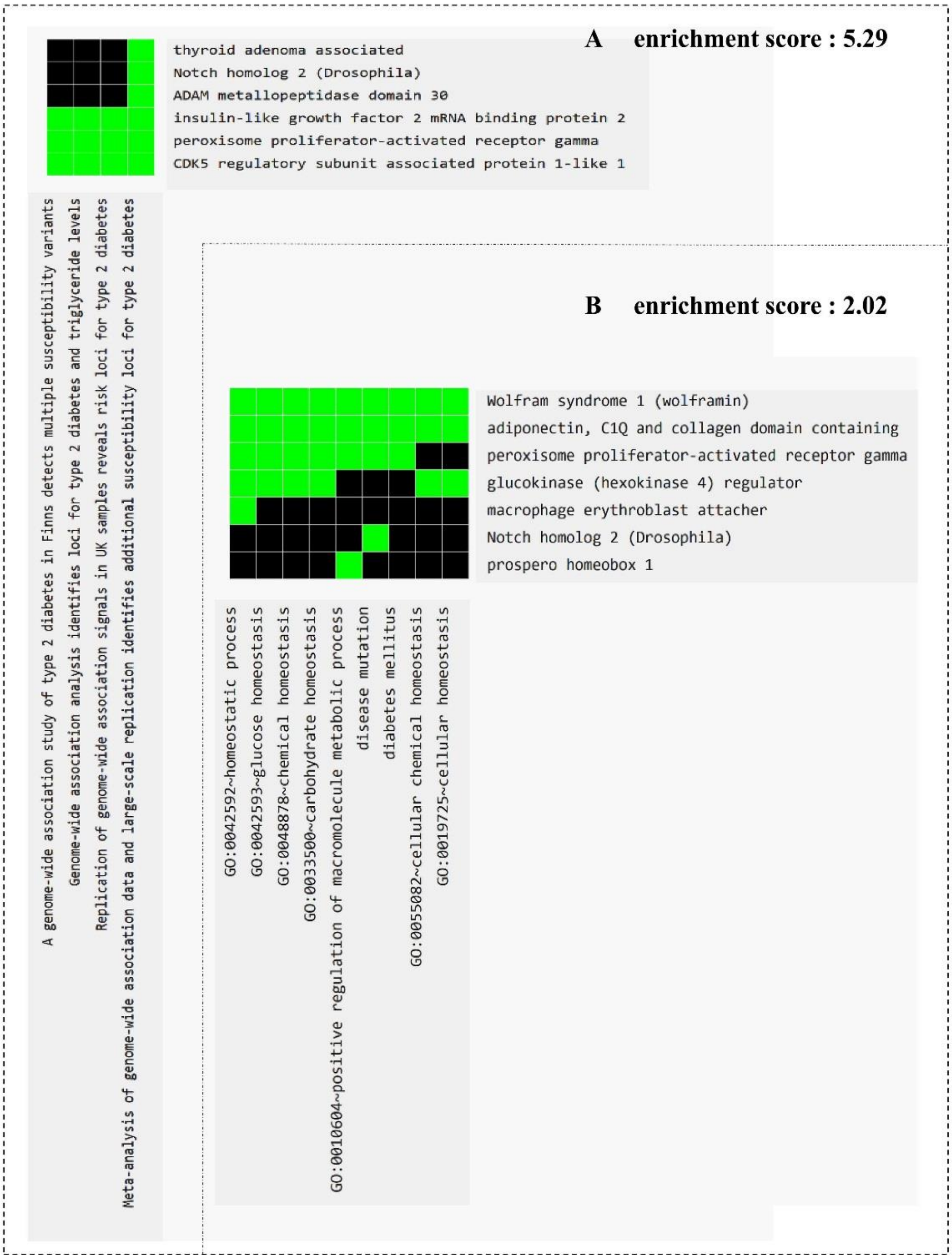

Functional annotation of genes associated with TSS SNPs through DAVID server. The results of top two clusters are displayed.

Figure S2 distributions of all LD SNPs reside in different colocalization histone marks.

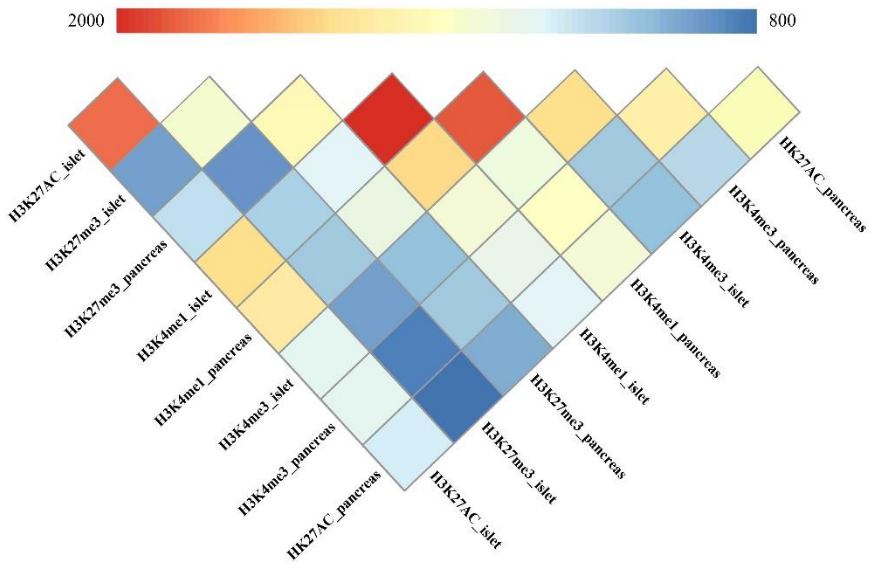

Very similar with the distributions of genotyped SNPs, H3K27me3 marks broadly harbored less overlapped SNPs with the activation marks.

Table S1 the information of six-five known risk SNPs in type2 diabetes.

https://github.com/LoopGan/supplementary-files-of-T2D-risks-estimation/blob/master/table%20S1.docx

The details of 65 T2D related SNPs (reference genome: Hg 19).

Table S2 the active SNPs and motifs obtained from HOMER and FIMO on whole genome.

https://github.com/LoopGan/supplementary-files-of-T2D-risks-estimation/blob/master/table%20S2.xlsx

The SNPs involved in binding events and motifs affected by SNPs in silico search by integrating results from HOMER and FIMO.

Table S3 effects of TSS SNPs on TFs result from motifbreakR based on HOMER motifs.

https://github.com/LoopGan/supplementary-files-of-T2D-risks-estimation/blob/master/table%20S3.xlsx

The detailed results of motifbreakR in analyzing TSS SNPs by using HOMER motifs.

Table S4 effects of TSS SNPs on TFs result from motifbreakR based on JASPAR motifs.

https://github.com/LoopGan/supplementary-files-of-T2D-risks-estimation/blob/master/table%20S4.xlsx
The detailed results of motifbreakR in analyzing TSS SNPs by using JASPAR motifs.

Table S5 a summary of genotyped TSS SNPs affect bindings.
https://github.com/LoopGan/supplementary-files-of-T2D-risks-estimation/blob/master/table%20S5.xlsx
TSS SNPs have strong effects on bindings and their related genes.

Table S6 a summary of analyses on Histone SNPs may affect regulations.
https://github.com/LoopGan/supplementary-files-of-T2D-risks-estimation/blob/master/table%20S6.xlsx
SNPs meet three conditions in detecting putative risk SNPs in histone marks, those meet any of three conditions are used in logistic regression model and in generating genotype score.

Table S7 a summary of Histone SNPs involved in previous GWA studies and eQTL analyses.
https://github.com/LoopGan/supplementary-files-of-T2D-risks-estimation/blob/master/table%20S7.xlsx
A summary result provided by Haploreg v4.1, which gives full information of concerned SNPs about any hits of eQTL in previous studies.
